# A polygenic basis for birth weight in a wild population of red deer (*Cervus elaphus*)

**DOI:** 10.1093/g3journal/jkad018

**Published:** 2023-01-18

**Authors:** Julie Gauzere, Josephine M Pemberton, Jon Slate, Alison Morris, Sean Morris, Craig A Walling, Susan E Johnston

**Affiliations:** Institute of Evolutionary Biology, University of Edinburgh, Edinburgh EH9 3FL, UK; AGAP, Université Montpellier, CIRAD, INRAE, Institut Agro, 34090 Montpellier, France; Institute of Evolutionary Biology, University of Edinburgh, Edinburgh EH9 3FL, UK; School of Biosciences, University of Sheffield, Sheffield S10 2TN, UK; Institute of Evolutionary Biology, University of Edinburgh, Edinburgh EH9 3FL, UK; Institute of Evolutionary Biology, University of Edinburgh, Edinburgh EH9 3FL, UK; Institute of Evolutionary Biology, University of Edinburgh, Edinburgh EH9 3FL, UK; Institute of Evolutionary Biology, University of Edinburgh, Edinburgh EH9 3FL, UK

**Keywords:** genome-wide association study, genomic prediction, GenPred‌, genomic relatedness, heritability, maternal effects, *Cervus elaphus*

## Abstract

The genetic architecture of traits under selection has important consequences for the response to selection and potentially for population viability. Early QTL mapping studies in wild populations have reported loci with large effect on trait variation. However, these results are contradicted by more recent genome-wide association analyses, which strongly support the idea that most quantitative traits have a polygenic basis. This study aims to re-evaluate the genetic architecture of a key morphological trait, birth weight, in a wild population of red deer (*Cervus elaphus*), using genomic approaches. A previous study using 93 microsatellite and allozyme markers and linkage mapping on a kindred of 364 deer detected a pronounced QTL on chromosome 21 explaining 29% of the variance in birth weight, suggesting that this trait is partly controlled by genes with large effects. Here, we used data for more than 2,300 calves genotyped at >39,000 SNP markers and two approaches to characterise the genetic architecture of birth weight. First, we performed a genome-wide association (GWA) analysis, using a genomic relatedness matrix to account for population structure. We found no SNPs significantly associated with birth weight. Second, we used genomic prediction to estimate the proportion of variance explained by each SNP and chromosome. This analysis confirmed that most genetic variance in birth weight was explained by loci with very small effect sizes. Third, we found that the proportion of variance explained by each chromosome was slightly positively correlated with its size. These three findings highlight a highly polygenic architecture for birth weight, which contradicts the previous QTL study. These results are probably explained by the differences in how associations are modelled between QTL mapping and GWA. Our study suggests that models of polygenic adaptation are the most appropriate to study the evolutionary trajectory of this trait.

## Introduction

Many quantitative traits appear to be subject to selection and yet show substantial levels of genetic variation in nature (Mousseau and Roff 1987; Kingsolver *et al*. 2001). Understanding how evolutionary forces act on genetic variation remains one of the most fundamental goals in evolutionary biology (Johnson and Barton 2005; Mitchell-Olds *et al*. 2007; Kruuk *et al*. 2008). To address this question, we need to understand the nature of quantitative trait variation, i.e. the number and identity of genes underlying trait variation, their physical location in the genome, and their interactions with each other. This detailed knowledge of genomic architecture can also help us to determine how a trait will respond to new selection pressures. For instance, a trait with an oligogenic architecture (i.e. affected by relatively few large effect loci) can evolve faster than a trait with a polygenic architecture (affected by many genes of small effect), but its genetic variation can also be quickly eroded with potential adverse effect on population viability in the long-term (Kardos and Luikart 2021). Yet, to date, there are still few descriptions of the genomic architecture of quantitative fitness-related traits in wild populations (for exceptions see Bérénos *et al*. 2015; Santure *et al*. 2015; Duntsch *et al*. 2020).

Since the development of molecular markers, quantitative genetic studies have sought to identify the individual loci responsible for trait variation (quantitative trait loci, QTL). Until recently, the main procedure for mapping genes was through “linkage mapping”, which conducted linkage analyses between markers and QTLs, usually performed using microsatellite markers and large half-sibling families, which limited its application in wild populations (Slate *et al*. 2010; but for an exception see e.g. Slate *et al*. 2002). With the genomic revolution, and reduced cost of sequencing and genotyping, genome-wide association studies (GWAS), which detect statistical associations between SNP markers and QTL that are sufficiently closely linked, have become increasingly accessible. This approach is more flexible than linkage mapping, but it requires much higher marker densities and sample sizes, which ultimately allow us to map QTLs with greater precision (Jensen *et al*. 2014; Gienapp *et al*. 2017). Most importantly, the development of GWAS in wild populations means that, in principle, we can now uncover the genomic basis of the large standing variation measured within natural populations. Nonetheless, this task remains challenging because a large proportion of phenotypic variation in nature is usually caused by non-genetic factors (e.g. due to environmental fluctuation; Kruuk 2004; Kruuk and Hadfield 2007), which needs to be accounted for in association analyses.

Until recently, genetic mapping and GWAS have been mainly performed in humans and model organisms. Their results strongly support the idea that most quantitative traits have a polygenic basis (Sella and Barton 2019). In humans, for instance, GWAS have discovered thousands of variants associated with complex traits and diseases (e.g. Goddard and Hayes 2009; Visscher *et al*. 2012; Yengo *et al*. 2018), each having a small effect on the trait’s value and explaining a very small fraction of the trait heritability. Long-term individual-based studies of wild vertebrates have greatly enhanced our understanding of the genetic basis of fitness-related traits in nature (Kruuk *et al*. 2008; Slate *et al*. 2010; Charmantier *et al*. 2014). Using these data sets, some genetic mapping studies have reported major effect loci, with QTLs explaining more than 20% of the heritability of quantitative traits (e.g. Slate *et al*. 2002; Poissant *et al*. 2012). However, these results could be an artefact due to the relatively small sample sizes available in non-model organisms (Slate 2013). Indeed, a well-known statistical bias is the inflation of QTL effect sizes when analysing moderate sample sizes (the Beavis effect; Beavis 1994). Replication of studies with larger data sets is therefore essential to validate the QTLs found in this literature.

The red deer population of the Isle of Rum, Scotland, has been intensively studied since 1972. Previous quantitative genetic studies have estimated the heritability of a variety of traits, as well as their association with fitness (Kruuk *et al*. 1999). Birth weight is a particularly interesting trait for evolutionary studies, as it is positively associated with juvenile survival and male reproductive success (Kruuk *et al*. 1999; Gauzere *et al*. 2022) and shows a moderate heritability, with h^2^ = 0.30 (0.23; 0.41) (Gauzere *et al*. 2020). We estimated that maternal effects, i.e. the influence of mother’s phenotype on the phenotype of her offspring (over and above the direct effect of genes inherited from her), largely contributed to this heritability value, these effects being mainly genetic in origin (Gauzere *et al*. 2020). Maternal genetic effects indeed explained half of this heritability value, the other half being due to the direct genetic effects. In a previous study, Slate *et al*. (2002) performed a QTL scan for birth weight using phenotypic, genotypic (90 microsatellites and 3 allozymes), pedigree and linkage map data in a kindred of 364 deer. The authors detected three potential QTL including a pronounced QTL on chromosome 21 explaining 29% of the genetic variance for birth weight. Here, we re-evaluate the genetic architecture of birth weight using a GWA approach, providing a more precise understanding of how genetic variation is distributed throughout the genome. The main drawback of this approach is that it did not allow us to decompose the direct and maternal genetic effects on birth weight, meaning that part of the genetic variance explored here is also due to maternal genetic effects.

In this study, we used data for more than 2,300 individuals and 39,000 SNPs evenly distributed across the genome to estimate the heritability of birth weight based on genomic relatedness. Next, we used GWA to search for genomic regions associated with birth weight. The model included a genomic relatedness matrix (GRM) to account for potential confounding effects of population structure (Price *et al*. 2006). Finally, we used the genomic prediction model BayesR (Moser *et al*. 2015) to analyse the distribution of SNP effect sizes and the proportion of variance explained by the SNPs on each chromosome. Our approach followed a similar recent study on the Soay sheep on St Kilda, which showed high accuracy to predict genomic estimated breeding values (GEBVs; Ashraf *et al*. 2021). Although genomic prediction analyses are usually used to estimate GEBVs, they can also be used to describe the genomic architecture of quantitative traits, such as the SNP-based heritability, the proportion of variance explained by large effect loci or by individual chromosomes. Here, we applied this approach for the first time in the wild red deer population.

## Material and methods

### Study population and SNP data set

The red deer living in the population in the North Block of the Isle of Rum, Scotland (57° 03′N, 06° 21′ W), have been intensively monitored since 1972. The calving period generally extends over 6 weeks, from mid-May to late June. Most calves born within the 12 km^2^ study area are caught soon after birth (often within 24 h), weighed, tagged, and sampled for DNA (via an ear punch). DNA is also routinely extracted from post-mortem tissues and cast antlers.

All sampled individuals within the study area have been genotyped at an attempted 51,248 SNP markers (Huisman *et al*. 2016) on the Illumina Cervine BeadChip using an Illumina iScan instument (Illumina Inc., San Diego, CA, USA). SNP genotypes were clustered using the Illumina GenomeStudio software and quality control was carried out using PLINK v1.9 with the following thresholds: SNP genotyping success >0.99, minor allele frequency >0.01 and individual genotyping success >0.99 (following Johnston *et al*. 2017). A linkage map specific to the Rum population is available, with 38,083 SNPs assigned to linkage groups corresponding to the 33 deer autosomes and X chromosome (Johnston *et al*. 2017). Estimated SNP positions are based on this linkage map. In total, *n* = 3,067 individuals were genotyped at 39,587 SNPs.

### Model of birth weight and GRM-based heritability

We analysed the weight (kg) of calves caught within 7 days of birth and born before August 1 (following Gauzere *et al*. 2020) and modelled it as a Gaussian trait. The model of birth weight accounted for the effect of calf sex, age at capture (hours), effects related to maternal condition, namely maternal age (years) and maternal reproductive status (as categorized in five levels), and the effect of birth location (categorized into six regions; see Gauzere *et al*. 2020). We also considered two random effects to account for the phenotypic variance in birth weight explained by variance in additive genetic effects (σ^2^_A_) and variance due to cohort effects (σ^2^_C_). Models of genetic architecture, especially genomic prediction models, do not easily accommodate different sources of genetic effects (e.g. maternal genetic effects). For this reason, we did not fit a maternal effect for birth weight here or in the following methods (more details can be found in the Supplementary Information SI). In a model omitting maternal effects, the maternal effect variance will mainly be confounded with additive genetic variance leading to inflated h^2^ estimates (Kruuk and Hadfield 2007). We know that maternal effect variance in birth weight is mainly due to genetic effects (Gauzere *et al*. 2020); maternal genetic effects explain 35% of the total phenotypic variance in birth weight and maternal environmental effects only explain 8% of the phenotypic variance. For this reason, we believe our models will not be biased by uncontrolled environmental effects.

We estimated the heritability of birth weight using an animal model and genomic information derived from a genome-wide relatedness matrix (GRM), namely a GRM-based heritability (h^2^_GRM_). The GRM was estimated using the *–make-grm* function in GCTA v1.90.2 (Yang *et al*. 2011). This GRM was adjusted to account for imperfect linkage disequilibrium (LD) between markers using the *–grm-adj 0* function. The estimated GRM was used to specify the covariance structure of the additive genetic effects in the birth weight model. The animal model was fitted using the R-package ASReml-R v3 (Gilmour *et al*. 2006).

### Genome-wide association study

A GWAS was performed to test for association between trait and SNP genotypes using the R-package RepeatABEL v1.1 (Rönnegård *et al*. 2016). First, we used the “prefitModel” function to fit the model of birth weight, with the fixed and random effects listed above, but without the SNP effects. We then used the “rGLS” function to test the effect of each SNP genotype on birth weight, accounting for the variance components previously estimated and the list of fixed effects. This model also accounted for population structure by fitting the GRM as a random effect. *P*-values for each SNP were computed using Wald statistics, distributed as χ^2^ with 1 degree of freedom. We divided these statistics by the genomic inflation factor λ to account for potential inflation due to population structure not captured by the GRM, where λ is defined as the median observed χ^2^statistic divided by the median χ^2^ statistic as expected from a null distribution (Devlin and Roeder 1999). A previous study by Johnston *et al*. (2018) on this dataset established the genome-wide significance threshold at a *P-value* of 1.42 × 10^−6^ after correcting for multiple testing and accounting for nonindependence due to linkage disequilibrium between SNP markers. In total, we analysed *n* = 2,317 calves.

### Estimation of SNP effect sizes and chromosome partitioning

The BayesR method implemented in the BayesR v0.95 software package (Moser *et al*. 2015) was used to infer the phenotypic variation explained by SNPs in different effect size groups and their contribution per chromosome. This method also estimated the SNP-based heritability (h^2^_SNP_). The SNP-based heritability differs from the GRM-based heritability as it estimates the proportion of variance accounted for by linear regression of a set of genotyped SNPs on the trait value (de los Campos *et al*. 2015). BayesR models SNP effects as a number of distributions of different effect sizes, including one of zero effect. Here, we ran the model with four distributions of effect size of 0, 0.0001, 0.001, and 0.01 (as a proportion of the phenotypic variance). BayesR does not allow for the inclusion of any fixed or random effects. Consequently, we first fitted the model of birth weight with lme4 (not considering any additive genetic effects) and we used the residuals of this model as phenotypes in Bayes R (following Ashraf *et al*. 2021; see Supplementary Information Part 1 for more information).

We first ran the model using a training population containing 95% of phenotyped and genotyped individuals (randomly selected). We then predicted genomic estimated breeding values (GEBVs) in the remaining 5%. We repeated this operation with a randomized 95% subset 10 times. The accuracy of the model was estimated as the Pearson correlation between the GEBVs and the observed phenotypic values, divided by the square root of the trait's heritability (estimated using an animal model with a GRM; h^2^_GRM_) (Ashraf *et al*. 2021). Finally, we used all the phenotypic and genomic information available to characterise the genomic architecture of birth weight using the estimated distribution of effect sizes. We estimated the proportion of variance explained by each chromosome using the allele frequencies and effect sizes of the mapped SNPs. We tested for the association between chromosome size and variance explained using a linear model. For polygenic traits, we expect the proportion of genetic variance explained by each chromosome to be directly related to its size (i.e. the number of genes it contains). The MCMC chains were run for a total of 120,000 iterations with a burnin of 20,000 and a thinning interval of 100, to sample 1,000 posterior samples for each parameter and individual.

### Estimation of effective population size and power calculation

The accuracy of association-based methods critically depends on the existence of linkage disequilibrium (LD) between causal loci and genetic markers. The effective population size (*Ne*) directly affects the pattern of LD across the genome. For this reason, genomic prediction shows low accuracy in wild populations with large effective population sizes (*Ne*) in comparison to domesticated species (Gienapp *et al*. 2019). Peters *et al*. (2022) have recently described the genome-wide LD pattern in the study population, and showed a relatively slow decay of LD across the genome. However, no study had yet used genomic data to estimate historical *Ne* in the red deer population. Here, we used the *SNeP* model developed by Barbato *et al*. (2015) to estimate *Ne* from LD information. This software considers squared Pearson’s product-moment correlation coefficient between pairs of loci to define LD. We fitted *SNeP* with default parameters.

We also performed a power analysis to evaluate the capacity of our GWA study to detect biologically meaningful QTLs (i.e. explaining ≥5% of genetic variance in birth weight). We conducted this analysis using an analytical method developed by Wang and Xu (2019), which uses as input the kinship matrix (GRM), the ratio of the additive genetic variance to the residual variance (estimated by the GWA model), and the sample size (*n* = 2,317). This calculation gives the power to detect association when the causative variant is typed. The power at the marker locus thus also depends on the coefficient of LD between the marker and causative variant (Pritchard and Przeworski 2001). Therefore, we adjusted the sample size to the effective sample size (n’) accounting for the linkage disequilibrium between neighbouring markers (r^2^= 0.2; Peters *et al*. 2022), so that n’ = 463.4.

## Results

### Analysis of power to detect QTL

We estimated that there is relatively good power to detect biologically meaningful QTLs (*power* = 0.72 for h^2^_locus_ = 0.05, and *power* ≥ 0.99 for h^2^_locus_ ≥ 0.10). Therefore, if birth weight has an oligogenic basis (as suggested by the previous QTL mapping study; Slate *et al*. 2002), we would be able to detect it. Based on genome-wide LD information, we estimated a relatively low effective population size of *Ne* = 175, which suggested that genomic prediction models are relevant to investigate and predict the genetic evolution of complex traits in the study populations.

### GRM-heritability and GWAS

The variance partitioning approach using an animal model and a GRM showed that birth weight had a significant and moderate heritability with h^2^_GRM_ = 0.38 (0.32; 0.44) (point estimate and 95% confidence intervals). This GRM-based heritability is very similar to a pedigree-based one with a model also omitting maternal effects (h^2^_PED_ = 0.45; see Supplementary Information, Part 1).

**Fig. 1.**
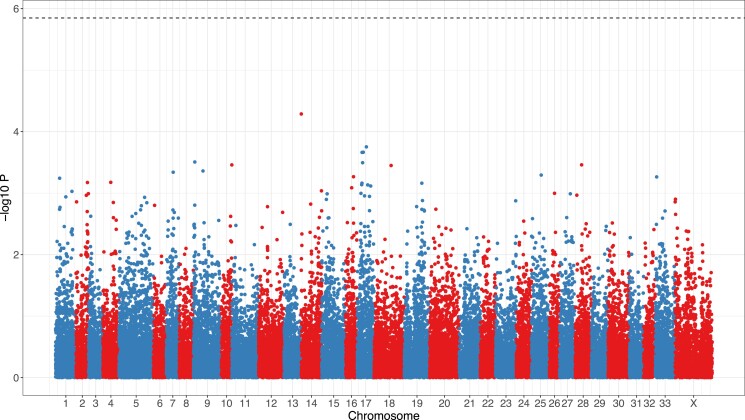
Manhattan plot for the association between birth weight and SNPs. Top dashed line: significance threshold equivalent to α = 0.05. Points are coloured by chromosomes (blue: odd numbers; red: even numbers). We only show results for the SNPs with known map positions. Previous potential QTLs found by Slate *et al*. (2002) were on chromosomes 12, 14 and 21 but not mapped with sufficiently good precision to be represented here.

**Fig. 2.**
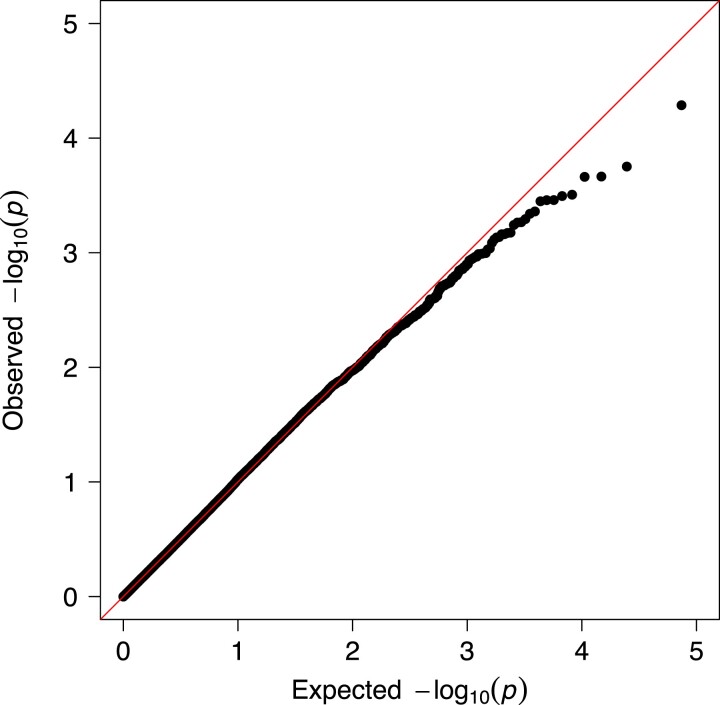
Quantile-quantile (Q-Q) plot of GWAS *P*-values for birth weight (shown in the Manhattan plot). This is a graphical representation of the deviation of the observed *P*-values from the null hypothesis. We found no *P*-values larger than expected under the null hypothesis (there are not points above the 1:1 diagonal).

The GWAS revealed a flat association landscape with no SNP significantly associated with birth weight (Fig. 1). The Q-Q plot showed that the *P*-values from the model were distributed as expected under a null model, and no obvious outliers were detected (Fig. 2). The genomic control inflation parameter was moderate, with λ = 1.34, and was used to adjust our test statistics to properly account for population structure. The top 10 SNPs with the lowest *P*-values are listed in Supplementary Table 1.

### Genomic prediction accuracy, distribution, and quantification of SNP effect sizes

**Fig. 3.**
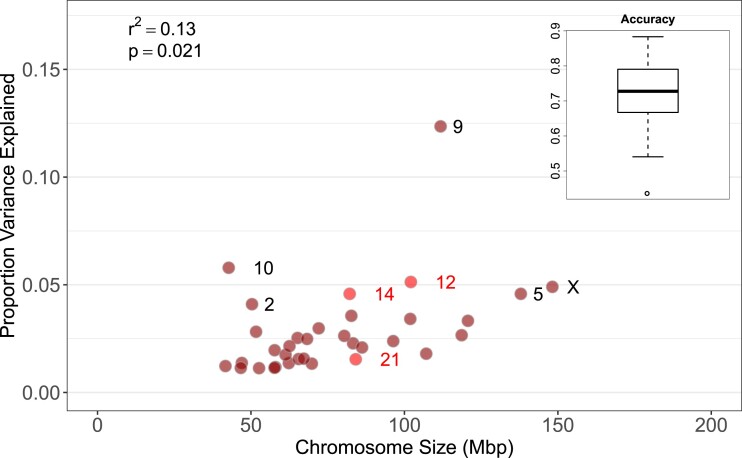
Chromosome partitioning from the genomic prediction analysis. The proportion of additive genetic variance explained by each chromosome is slightly positively correlated with their size. The genomic prediction model has a high accuracy to predict the breeding values for birth weight, with a mean accuracy of 0.71 (using h^2^_SNP_ = 0.38). The previous QTLs reported by Slate *et al*. (2002) were found on chromosomes 21, 12 and 14 (in red), which respectively explain here 1.5, 5.1 and 4.6% of the genetic variance.

The genomic prediction model had a high accuracy to predict the genomic estimated breeding values (GEBVs) in the red deer population. This is an important finding, as models such as BayesR have not yet been tested and validated on many wild animal data sets (see e.g. Gienapp *et al*. 2019; Ashraf *et al*. 2021). The mean model accuracy was 0.71, with a minimum of 0.44 and a maximum of 0.88 (Fig. 3). The estimated heritability using the genomic prediction model was h^2^_SNP_ = 0.38 (0.26; 0.49) (95% credible intervals), very similar to the GRM- and pedigree-based heritabilities estimates above.

We found a positive correlation between the chromosome size and the proportion of additive genetic variance explained by each chromosome (Fig. 3). Chromosome 9 explained most variation, but this was nevertheless a very low percentage of the total genetic variation (<12.5%). Most SNPs had no effect on birth weight and only 5,805 SNPs were predicted to have a non-zero effect on birth weight. The largest proportion of genetic variance is explained by loci with very small effect sizes (Table 1 and Supplementary Table 2). Altogether, results from the genomic prediction model are suggestive of a highly polygenic architecture. Nonetheless, one should be cautious when interpreting these estimates, especially regarding the number of SNPs with small effect sizes and proportion of variance explained by these SNPs, which are estimated with a very large uncertainty (Table 1). As also noted by Ashraf *et al*. (2021), the relatively small sample sizes used in studies in natural populations probably make it difficult to distinguish between zero effect and small effect loci.

**Table 1.** Number and effect sizes of SNPs contributing to phenotypic variation.

	Mean estimate	Lower CI	Upper CI
V_A_	0.456	0.30	0.66
V_E_	0.757	0.67	0.85
N_SNP_	5,805	1,179	9,432
N_SNP_0_	33,782	30,155	38,408
N_SNP_0.0001_	5,402	260	9,392
N_SNP_0.001_	392	117	951
N_SNP_0.01_	10	0	37
PGV_0.0001_	0.54	0.03	0.94
PGV_0.001_	0.39	0.02	0.93
PGV_0.01_	0.07	0.00	0.26

Results from the genomic prediction model. N_SNP_ provides the number of non-zero SNPs and N_SNP_X_ the number of SNPs in each effect size groups X. PGV is the proportion of genetic variance assigned to the three non-zero effect size distribution (sum equals 1).

## Discussion

Two decades of genetic mapping in wild populations have provided contrasting results about the genetic architecture of fitness-related traits (Slate *et al*. 2010; Jensen *et al*. 2014). Some of the earlier works, which showed apparently large effect loci using QTL mapping techniques, need to be revaluated using high-density genome-wide markers. In this study, we used two different genomic approaches to describe the genetic architecture of birth weight in a natural population of red deer, a trait known to be under strong positive selection (Kruuk *et al*. 1999; Gauzere *et al*. 2022). Both approaches demonstrate that this trait is heritable and has a polygenic architecture. Pedigree and GRM-based heritability estimates were very similar, indicating that the genotyped SNPs are in sufficiently high linkage to represent the recombination events in the study population (as also found by Peters *et al*. 2022). Additionally, Peters *et al*. (2022) recently showed that the linkage disequilibrium among the SNPs present on the red deer SNP chip is maintained over a distance of up to 1Mb. Such linkage is essential in mapping studies and suggests that we should be able to detect major QTLs, although not necessarily locate them with great precision. These results indicate that pedigree-free approaches offer a promising prospect for measuring the heritability of fitness-related traits in the wild, even when using relatively small sample sizes (Bérénos *et al*. 2014; Perrier *et al*. 2018).

Three different results provide evidence that birth weight has a highly polygenic genetic architecture: (1) there were no significant SNP associated with birth weight in the GWAS, (2) in the BayesR model, most variation was explained by loci with very small effect sizes and (3) in the same analysis, the variance explained by each chromosome scaled with chromosome size. These findings are in line with a number of recent genomic studies usually showing flat association landscapes (e.g. Hansson *et al*. 2018; Duntsch *et al*. 2020; Peters *et al*. 2022), but contradict a previous study on the genetic architecture of birth weight in the study population, which reported a major effect QTL on chromosome 21 (Slate *et al*. 2002). A similar scenario applies in the great reed warbler system, in which a large effect QTL for wing length found using linkage mapping was not found using GWAS, albeit with a very small sample size for GWAS (181 individuals; Hansson *et al*. 2018). Here, we confirmed that, with the sample size and number of markers used in this study, we have enough power to detect moderate to large effect size loci affecting birth weight if such major QTLs existed; we can thus confidently exclude this hypothesis.

Mapping studies using small sample sizes can overestimate the effect size of significant QTLs and/or identify false positive QTLs due to the Beavis effect (Slate 2013). The Beavis effect has a biological explanation: fewer recombination events are represented in small sample sizes, hence multiple loci that affect a trait can be misidentified as single QTL with a large effect (Josephs *et al*. 2017). This bias likely affected the results of the previous QTL study that used a sample size of about 350 individuals. However, if the previous results were entirely due to the Beavis effect and the co-segregation of multiple QTLs, we would have expected to detect some signal at the chromosome 21. On the contrary, we found that chromosome 21 is among those that explains the smallest amount of variation in birth weight (1.5%). Consequently, poor resolution does not explain the large effect QTL previously reported. It is most likely that the result by Slate *et al*. (2002) was due to the fact that microsatellite-based and pedigree-based QTL mapping are more sensitive to spurious associations (Hansson *et al*. 2018) and that lower significance thresholds were used. Moreover, the previous analysis was conducted within one specific kindred in the red deer population. A spurious association could therefore have emerged if related individuals in that lineage shared unique alleles at loci on chromosome 21 and also had higher/lower birth weight. Because GWAS and genomic prediction models are fundamentally different methods from QTL mapping that look at the statistical association between genotype and phenotype and do not focus on the co-segregation of markers in a pedigree, they are less sensitive to this bias. Finally, population structure and hidden family structure can also cause false positives in mapping studies (Price *et al*. 2006; Wood *et al*. 2014). GWAS uses a mixed model framework that is flexible enough to allow the inclusion of a genomic relatedness matrix to account for this structure. We also corrected our statistics by the inflation factor to avoid detecting SNPs erroneously associated with birth weight.

When analysing natural populations, one of the biggest challenges is to control for potential environmental covariates that might affect trait variation. Indeed, such covariates might buffer the signal of genotype-phenotype association, which might explain why plant GWAS performed in controlled environmental conditions are usually able to map larger effect QTLs (Josephs *et al*. 2017). Here, we controlled for the major environmental effects and covariates known to affect birth weight. We also know that maternal effects are an importance source of phenotypic variance in birth weight (35% of phenotypic variance explained; Gauzere *et al*. 2020). However, the approach used did not allowed us to decompose the maternal effect variance from direct genetic variance (see Supplementary Information Part 1 for more details). This is because almost all the maternal effect variance is due to maternal genes (Gauzere *et al*. 2020). Decomposing the genetic architecture of direct and maternal genetic effects is a daunting task that requires statistical cross-fostering (Wolf and Cheverud 2012) or modelling the effect of transmitted and non-transmitted alleles (Kong *et al*. 2018), methods that have only been applied in model systems or very large human datasets. Because we found a polygenic architecture for birth weight, we do not believe that decomposing these effects will provide more information than the current analysis. However, one has to keep in mind that part of the genetic variance explored here is due to maternal genetic effects.

Our results highlight the potential of genomic prediction models to study the trait architecture and evolution in this wild population. Indeed, we report a high accuracy to predict GEBVs that is comparable to that found in a similar wild study system, the Soay sheep of St Kilda (Ashraf *et al*. 2021), and in plant and animal breeding (e.g. de los Campos *et al.* 2013; Bhat *et al*. 2016). We know that the accuracy of genomic prediction depends, among other things, on the effective population size (*Ne*), which directly affects LD patterns across the genome (Visscher *et al*. 2006; Hill and Weir 2011). Here, we study an island population with a relatively small *Ne* (*Ne* = 175), which explains this high accuracy in comparison to other wild populations (e.g. in birds; Gienapp *et al*. 2019). Most importantly, our model is flexible enough to capture different genetic architectures, as it models SNP effects as a mixture of distributions describing large and small effect sizes. Nonetheless, further characterization of the architecture of a polygenic trait such as deer birth weight would require a much larger data set than the one used here to disentangle small effect sizes from zero effect.

This study uses one of the largest datasets in a wild population, with ∼2,300 individuals phenotyped and genotyped, to explore the genetic architecture of a fitness-related trait. This knowledge of the underlying genetic architecture of quantitative traits is most important to understand the origin of genetic variance and how it evolves. According to theory, the evolution of birth weight, a trait with a highly polygenic architecture, will be driven by subtle changes of allelic frequencies at many QTLs and by covariance among QTLs (Le Corre and Kremer 2003). This polygenic architecture should counteract the loss of genetic variation due to natural selection (Sella and Barton 2019). The results found here suggest that models of polygenic adaptation that explicitly model the genome-wide covariance between allele frequency changes in temporal or spatial data are the most appropriate to measure the genomic imprint of natural selection (see e.g. Buffalo and Coop 2020).

## Supplementary Material

jkad018_Supplementary_Data

## Data Availability

All the genomic and phenotypic data used in this paper have been deposited on figshare, https://doi.org/10.6084/m9.figshare.21842130. Supplemental material available at G3 online.
