# Supplementary material for: A polygenic basis for birth weight in a wild population of red deer (Cervus elaphus):
Source: G3 (Bethesda). 2023 Jan 18;13(4):jkad018. doi: 10.1093/g3journal/jkad018 (PMC10085764; doi:10.1093/g3journal/jkad018)
Supplement: jkad018_Supplementary_Data [file jkad018_supplementary_data.docx]

**Supporting Information for ‘A polygenic basis for birth weight in a wild population of red deer (*Cervus elaphus*)’**

**Part S1. Models of genetic architecture with and without maternal effects**.

In red deer, maternal effects are known to affect birth weight; they explain about 35 % of the phenotypic variance in this neonatal trait (Gauzere et al. 2020). In this study, we used a genomic prediction model (BayesR) to investigate the genetic architecture of birth weight. This approach is not flexible enough to allow the inclusion of fixed and/or random effects other than the SNP effects. Therefore, we used a two-step approach that consisted of (1) fitting the effect of potential environmental covariates on birth weight and (2) using the residuals of this model as phenotypes in BayesR. In step (1), we initially also accounted for variation due to maternal effects and cohort effects by fitting them as random effects using a linear mixed model (R-package ‘lme4’). However, by fitting maternal effects, we removed almost all the direct additive genetic variance in birth weight due to the calf’s own genes. Using the BayesR software, we then estimated h^2^_SNP_ = 0.054, instead of h^2^_SNP_ = 0.38 using a model without maternal effects, which is much closer to the pedigree-estimated total heritability, h^2^_TOT_ = 0.31 (Gauzere et al. 2020). This result is probably due to the fact that variance in maternal effects is mainly genetic (Gauzere et al. 2020) and partly confounded with variance due to direct genetic effects. Therefore, for the main analysis reported in the MS, we decided to consistently use models that excluded maternal effects. This means that the models presented in the main text capture the *total* genetic variance in birth weight, i.e., the variation due to both direct and maternal genetic effects.

We also fitted a GWA model that accounted for variance in maternal effects on offspring weight to check whether this would affect our results. As illustrated in Fig S1, we found a flat association landscape with no SNP significantly associated with birth weight, similar to Fig. 1 in the main text.

We also estimated the pedigree- and GRM-based heritabilities using models with and without maternal effects. Using models omitting maternal effects, we found a slight discrepancy between the two estimates, with h^2^_PED_ = 0.45 (with a standard error SE of 0.039) and h^2^_GRM_ = 0.38 (SE 0.032). Such models lead to overestimation of the variance due to direct genetic effects, but this bias was larger for h^2^_PED_ than h^2^_GRM_, probably because GRM contains more information to accurately estimate this parameter than the pedigree. When maternal effects were included, the two estimates were very similar, with h^2^_PED_ = 0.16 (SE 0.036) and h^2^_GRM_ = 0.17 (0.027).

**Figure S1**: Manhattan plots for the association between birth weight and SNPs using a model that included maternal effects. Top dashed line: significance threshold equivalent to α = 0.05. Points are coloured by chromosomes (blue: odd numbers; red: even numbers).
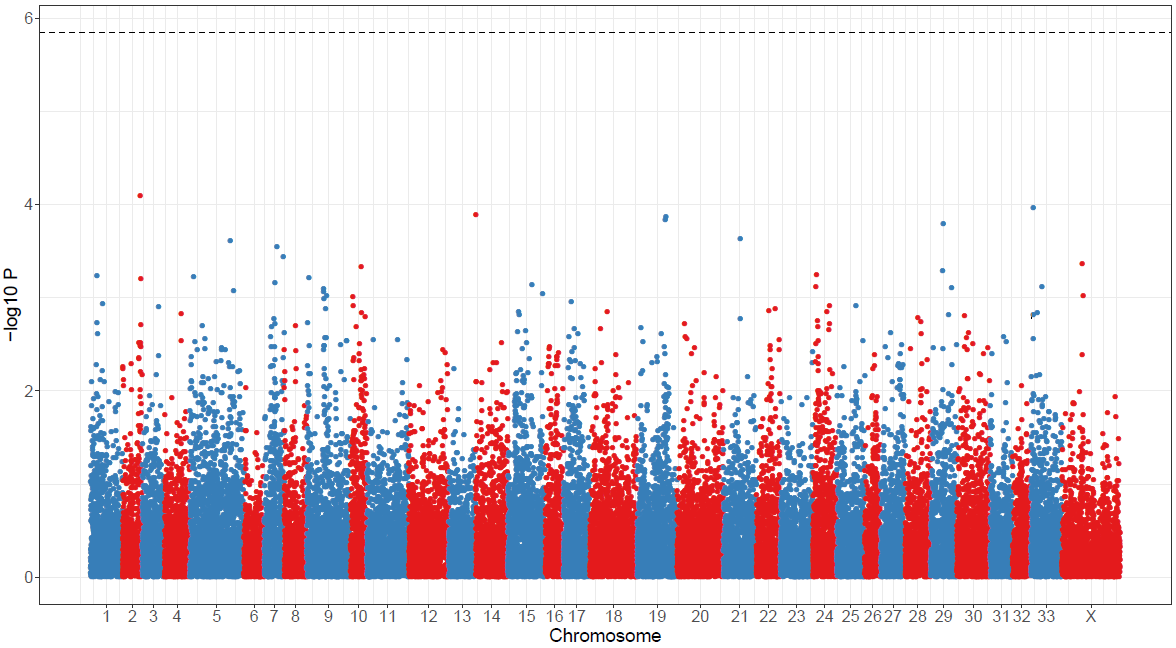


**Table S1**: Summary information for the top 10 SNPs with the lowest p-values for their association with birth weight from the GWAS analysis. The ‘Pc1df’ column provides the p-value corrected for the inflation factor λ.

| **CHR** | **Position** | **SNP.Name** | **effect size** | **SE effect size** | **Pc1df** |
| --- | --- | --- | --- | --- | --- |
| *14* | *1124027* | *cela1_red_16_1087419* | *0.21400* | *0.03343* | *0.00005* |
| 17 | 37231561 | cela1_red_6_37310659 | 0.24266 | 0.04092 | 0.00018 |
| 17 | 24918351 | cela1_red_6_24997449 | 0.35164 | 0.06011 | 0.00022 |
| 17 | 20138871 | cela1_red_6_20217969 | 0.28595 | 0.04890 | 0.00022 |
| *9* | *6318552* | *cela1_red_7_6297184* | *0.19294* | *0.03384* | *0.00031* |
| 17 | 22270930 | cela1_red_6_22350028 | 0.77175 | 0.13562 | 0.00032 |
| 28 | 27016058 | cela1_red_9_26401576 | -0.18753 | 0.03315 | 0.00035 |
| 10 | 42689353 | cela1_red_25_42724436 | -0.51469 | 0.09099 | 0.00035 |
| 18 | 68321580 | cela1_red_4_68350340 | 0.94808 | 0.16788 | 0.00036 |

**Table S2:** Summary information for the top 10 SNPs by variance explained from the genomic prediction model. PIP are the posterior inclusion probabilities of the SNP for each of the 4 distributions (with effect size groups 1:0, 2: 0.0001, 3: 0.001, 4: 0.01).

| **CHR** | **Position** | **SNP name** | **PIP1** | **PIP2** | **PIP3** | **PIP4** | **Effect size** |
| --- | --- | --- | --- | --- | --- | --- | --- |
| *9* | *6318552* | *cela1_red_7_6297184* | *0.613* | *0.141* | *0.116* | *0.13* | *-0.018622* |
| 9 | 9264710 | cela1_red_7_10995860 | 0.601 | 0.155 | 0.135 | 0.109 | -0.01670565 |
| *14* | *1124027* | *cela1_red_16_1087419* | *0.634* | *0.183* | *0.106* | *0.077* | *-0.0128306* |
| 10 | 28147512 | cela1_red_25_28182595 | 0.658 | 0.156 | 0.099 | 0.087 | -0.01355471 |
| 10 | 28145924 | cela1_sika_25_28181007 | 0.672 | 0.14 | 0.114 | 0.074 | -0.01350112 |
| 12 | 15296948 | cela1_red_10_14997406 | 0.664 | 0.157 | 0.115 | 0.064 | 0.01111467 |
| 2 | 41926862 | cela1_red_29_43249226 | 0.698 | 0.154 | 0.095 | 0.053 | 0.009840739 |
| X | 9890928 | cela1_red_x_62846435 | 0.664 | 0.169 | 0.128 | 0.039 | -0.009501642 |
| 4 | 34830380 | cela1_red_18_34180996 | 0.722 | 0.15 | 0.082 | 0.046 | 0.008222215 |
